# Soil Nutrient Assessment for Urban Ecosystems in Hubei, China

**DOI:** 10.1371/journal.pone.0075856

**Published:** 2013-09-27

**Authors:** Zhi-guo Li, Guo-shi Zhang, Yi Liu, Kai-yuan Wan, Run-hua Zhang, Fang Chen

**Affiliations:** 1 Key Laboratory of Aquatic Botany and Watershed Ecology, Wuhan Botanical Garden, Chinese Academy of Sciences, Wuhan, China; 2 Wuhan Academy of Agriculture Science and technology, Vegetable Research Institute, Wuhan, China; Roehampton university, United Kingdom

## Abstract

Recent urban landscape vegetation surveys conducted in many cities in China identified numerous plant nutrient deficiencies, especially in newly developed cities. Soil nutrients and soil nutrient management in the cities of Hubei province have not received adequate attention to date. The aims of this study were to characterize the available nutrients of urban soils from nine cities in Hubei province, China, and to assess how soil nutrient status is related to land use type and topography. Soil nutrients were measured in 405 sites from 1,215 soil samples collected from four land use types (park, institutional [including government building grounds, municipal party grounds, university grounds, and garden city institutes], residential, and roadside verges) and three topographies (mountainous [142–425 m a.s.l], hilly [66–112 m a.s.l], and plain [26–30 m a.s.l]). Chemical analyses showed that urban soils in Hubei had high pH and lower soil organic matter, available nitrogen (N), available phosphorus (P), and available boron (B) concentrations than natural soils. Nutrient concentrations were significantly different among land use types, with the roadside and residential areas having greater concentrations of calcium (Ca), sulfur (S), copper (Cu), manganese (Mn), and zinc (Zn) that were not deficient against the recommended ranges. Topographic comparisons showed statistically significant effects for 8 of the 11 chemical variables (p < 0.05). Concentrations of N, Ca, Mg, S, Cu, and Mn in plain cities were greater than those in mountainous cities and show a negative correlation with city elevation. These results provide data on urban soils characteristics in land use types and topography, and deliver significant information for city planners and policy makers.

## Introduction

Urbanization has been an especially rapid process in developing countries such as China. By 2050, more than half of the world’s population will probably reside in cities, and almost all population growth will be in cities in developing countries [1]. Consequently, the expansion of urban construction will intensify as will the potential for emission of greenhouse gases [2].

Urban greenland is an important and essential component of urban construction that not only filters harmful substances, improves micro-climates, and reduces noise, but also improves the aesthetic value of the city, propagates progressive city culture, and reduces greenhouse gas emissions [3]. Many cities extend greenland areas within their limited urban space and improve the quality of urban greenland areas to build ‘green’ or ‘low-carbon’ cities. However, after extensive ‘green city’ construction, it has been found that garden plants fail to form the expected ‘garden’ landscape, and some plants die from wilt disease a few years later after transplanting [4]. These problems have become the main limiting factor for urban landscape construction in many cities in China.

Poor soil condition may be one of the main reasons for unhealthy urban garden plants [5]. Urban soils are often dramatically altered by human activities such as construction, compaction, degradation, land filling, and mixing. Topsoil is usually filled with stones, construction rubble, bricks, and other building materials, which likely contribute to poor soil fertility [6] and are not within the recommended nutrient requirements range [7-9]. However, to date there is little consensus among researchers on this issue. In Hong Kong, Jim [11] found that two-thirds of sampled soils had an elevated pH up to 8.68 and low organic carbon at 0.88%. Zhang et al. [10] reported that available nitrogen (N) (26.5 mg kg^-1^) and available phosphorus (P) (19.7 mg kg^-1^) in urban soils were significantly lower than the recommended range (50–100 mg kg^-1^ for N and 30–60 mg kg^-1^ for P) of non-urban soils [7-9]. White and McDonnell [12] also found that the net N mineralization rate in urban forest soils in New York City was significantly lower than that in rural forest soils. In contrast, Pouyat et al. [8] found, although a subset of soil chemical and physical properties varied considerably among different land use and cover types in Baltimore, MD, USA, most sites had sufficient available soil nutrients to support plant growth.

Land use and topography are important factors affecting soil nutrients [8], but knowledge on these factors in urban areas is limited. The aim of our study was to characterize the available nutrients of urban soils from nine cities in Hubei province, China, and to assess how soil nutrient status was related to land use type and topography. It was hypothesized that the status of available nutrients in urban soil would vary significantly with land use and topography. These baseline findings could provide practical information for making better urban soil management strategies in Chinese cities.

## Materials and Methods

### Ethics Statement

This study has been approved by the City Planning Office Construction Department of Hubei province, which takes care of the planning of urban construction in Hubei province. All the data in this study can be published and shared.

### Study area

Hubei Province (Latitude 29° 05’–33°20′, Longitude 108° 21′–116°07′) is located in central China, with an area of 185,900 km^2^ and a population of 61.7 million in 2010 [13]. Hubei has always been the waterway and overland transportation hub of central China [13]. Landforms in this region are a diverse arrangement of mountains, hills, and plains. Mountainous area occupies ~55.5% of the total provincial area, with 24.5% classed as hilly, and 20% classed as plains [14]. Average annual rainfall is 800–1600 mm and average annual temperature is 15–17°C. Because of these different landforms, there are large differences in temperature and rainfall between the mountainous and plain areas with plains usually having more rain and higher temperatures in summer [14].

### Study sites

Nine cities in Hubei, including three mountainous cities (Yichang, Shiyan and Enshi, with an average elevation of 276 m); three hilly cities (Jingmen, Suizhou and Xiangfan, with an average elevation of 83 m); and three plain cities (Jingzhou, Wuhan and Xiaogan, with an average elevation of 28 m) were selected (Table 1). Most of the sampled soils at each site were yellow-brown, derived from water-transported (alluvium) or wind-blown (loess) material, yet some have been formed by in-situ weathering of rocks (carbonate rocks and clastic rocks including shale, silt limestone, and pelitic siltstone). The urban greenland of each city was classified into four land use categories (Table 2) based on satellite remote-sensing imagery (10 August, 2010). The park areas were the least disturbed with a natural soil material but a small amount of building rubble incorporated. The soil at the institutional areas was moderately disturbed with remnants of building rubble and natural substrates and typical managed vegetation. By contrast, the residential areas were more heavily disturbed with additions of building rubble and domestic sewage. The soil in the roadside was also the most heavily disturbed and often compacted by humans and vehicles. The mixed substrates, with transferred natural substrates, remnants of building rubble, pitch, and domestic waste, were the parent material for soil development at roadside sites.

**Table 1:** Descriptions of the nine study cities [14].

Site	Landforms	Elevation (m)	Average annual precipitation (mm)	Average annual temperature (°C)	Soil types	Population size (thousand)	City area (km^2^)	Soil sampling numbers
Shiyan	Mountain	263	800	13.1-16	Yellow brown soil	310	62	42
Enshi	Mountain	425	1000-1200	13.4-16.3	Yellow brown soil	800	80	39
Yichang	Mountain	142	992	13.1-18	Yellow brown soil	557	92	45
Jingmen	Hill	112	949	16.1	Paday soil	680	50	39
Suizhou	Hill	66	865-1070	15.5	Yellow brown soil	650	43	39
Xiangfan	Hill	71	878	15.1-16.9	Yellow brown soil	2230	107	42
Jingzhou	Plain	30	1100-1300	15.9-16.6	Yellow brown soil	1140	66	42
Wuhan	Plain	27	1100	15.8-17.5	Yellow brown soil	5170	500	78
Xiaogan	Plain	26	1112	15.8	Paday soil	950	33	39

**Table 2:** Characteristics of the four land use types.

Land use type	Number of samples	Parent material	Vegetation	Management	Disturbance degree [15]
Park	87	Natural substrates, small building rubble	Unmanaged and managed trees, shrubs, herbaceous	Irrigation, no fertilizer	Little disturbance
Institution	131	Transferred natural mixed substrates, building rubble, bricks, cement	Managed trees, shrubs, herbaceous	Irrigation, no fertilization, human trampling	Moderate disturbance
Residential	98	Transferred natural mixed substrates, building rubble, bricks, cement, domestic garbage	Managed trees, shrubs, herbaceous	Irrigation, no fertilizer, human trampling, pet feces, sewage	Heavy disturbance
Roadside	95	Transferred natural mixed substrates, building rubble, bricks, cement, domestic waste, plastics pitch	Managed trees, shrubs, herbaceous	Irrigation, no fertilizer, human trampling, mechanical compression	Heaviest disturbance

### Soil sampling and chemical analysis

Soil sampling was conducted from August 10–29th in 2010. Nine to 20 sites in each greenland land use type of each city were selected depending on city size. Within each site, three 3 × 5 m replicate subplots were randomly established. Soil samples from the surface layer (0–20 cm), with a composite of 10 subsamples, were taken with a 2-cm diameter stainless steel sample auger at each subplots. A total of 1215 soil samples were taken from 405 sites. In the laboratory the samples were air dried and sieved to 1 and 2 mm. Soil pH was measured in a soil-to-water suspension ratio of 1:1. Soil organic matter (SOM) was determined using the K_2_CrO_7_-H_2_SO_4_ oxidation method, available N was determined by the Alkali N-proliferation method. Available calcium (Ca), magnesium (Mg), potassium (K), P, sulfur (S), iron (Fe), copper (Cu), manganese (Mn), zinc (Zn), and boron (B) were determined using ASI methods [7,16] modified by Agro Services International, Inc. (ASI). Three soil extraction solutions were used to extract soil available nutrients: (1) ASI soil extracting solution (0.25 mol L^-1^ NaHCO_3_, 0,01 mol L^-1^ EDTA, 0.01 mol L^-1^ NH_4_F, and 0.05 g L^-1^ Superfloc), used to measure available P with a UV-8500 spectrophotometer (Tianmei Co., Shanghai, China) and K, Fe, Cu, Mn and Zn with an AA-6300 atomic absorption spectrometer (Shimadzu, Kyoto, Japan); (2) ASI-KCl extraction, used to measure available Ca and Mg with an AA-6300 atomic absorption spectrometer (Shimadzu, Kyoto, Japan); and (3) ASI-Ca(H_2_PO_4_)_2_ extraction, used to measure available S and B with UV-8500 spectrophotometer (Tianmei Co., Shanghai, China).

### Data analysis

Variables were tested for normality (Kolmogorov–Smirnov) and for homogeneity of variances (Levene’s test). A two-way ANOVA using SPSS version 16.0 (SPSS Inc., Chicago, IL, USA) was performed for the effects of land use and topography types on each soil variable [17]. Differences between means were tested using Duncan’s multiple range at p < 0.05.

## Results

Urban soil pH ranged from 4.90 to 8.76, with a mean of 7.88 (Table 3). Only 8% samples fell below neutrality, all the rest were alkaline. The concentrations of SOM were very low, with a mean of 6.7 g kg^-1^. For most soils, SOM < 10 g kg^-1^ is considered low. As to available N and P, urban soils had relatively lower levels (31.5 and 28.3 mg kg^-1^, respectively) when compared with the normal range (50–100 mg kg^-1^ and 30–60 mg kg^-1^, respectively) of non-urban soils (Table 3). However, for available K, it is apparent that in many of the plots, Hubei urban soils have sufficient nutrient levels (125 mg kg^-1^). Compared with macronutrients, secondary nutrients (Ca, Mg, and S) did not show deficiency, and in the case of available Ca (2694 mg kg^-1^) were excessive. The micronutrients (Mn, Fe, and Zn) had sufficient or excessive nutrient levels to support plant growth. Mean urban soil B concentration was 0.86 mg kg^-1^ (within the common range of 0.6–1 mg kg^-1^); however, from the frequency distribution of B in the samples, 60% of samples fell below the critical value. Thus, most urban soils, especially in the plain cities, have inadequate soil B supply.

**Table 3:** Descriptive statistics of soil properties from the 0–20 cm layer in nine cities in Hubei province (n = 405).

Soil properties	Minimum	Maximum	SE	CV (%)	Mean	Recommended range^a^
pH	4.9	8.76	0.55	6.96	7.88	4-6
Organic matter, g kg^-1^	0.94	19.42	2.97	43.55	6.73	>10
N, mg kg^-1^	1.96	314.01	35.55	112.52	31.56	50-100
P, mg kg^-1^	1.2	385.85	43.72	111.43	28.33	30-60
K, mg kg^-1^	33.45	507.42	69.13	55.39	125.31	78-156
Ca, mg kg^-1^	892	6048	916	34	2694	300-1000
Mg, mg kg^-1^	26.25	830.1	139.16	57.26	244.35	100-500
S, mg kg^-1^	1.93	146.31	23.87	74.74	31.92	24-40
Fe, mg kg^-1^	5.03	149.74	21.21	64.37	32.94	20-30
Cu, mg kg^-1^	0.43	23.65	2.82	73.34	3.87	3.0-4.0
Mn, mg kg^-1^	1.71	146.44	20.38	84.82	23.9	6.0-15.0
Zn, mg kg^-1^	0.91	43.06	5.63	88.49	6.38	3.0-6.0
B, mg kg^-1^	0.01	5.33	0.88	101.8	0.86	0.6-1.00

a According to Portch and Hunter [7] and Whitcomb [9].

Table 4 shows the results of a two-way ANOVA for the effects of land-use and topography types on each soil variable. Significant differences in soil nutrients between four land use types were found (p < 0.05, Table 4). Concentrations of available Ca, S, and Cu in the roadside sites were significantly higher than those in the institutional sites, while available Mn in the park sites was much higher than that in residential sites (Table 5). Interestingly, for available soil N and K, no significant differences were found between the four land use types, but for available P, it was as much as 1.6 times greater in parks than in the residential sites (27.1 mg kg^-1^). Compared with the recommended range, soil P concentration in the residential sites was slightly lower than the critical level, while for N, the concentrations in all land types were significantly lower than the critical level. No significant differences in SOM were found among the four land use types, but the mean values in all types were significantly lower than the critical level.

**Table 4:** Summary statistics of a two-way ANOVA looking at the effects of land use and topography types on surface soil properties.

Main effects	df	pH		SOM		N		P		K		Ca		Mg	
		F	Sig.	F	Sig.	F	Sig.	F	Sig.	F	Sig.	F	Sig.	F	Sig.
Land use (A)	3	3.86	0.01	0.68	NS	0.33	NS	3.84	0.01	1.08	NS	3.24	0.03	1.82	NS
Topography (B)	2	8.41	< 0.01	10.25	0.01	18.34	< 0.01	1.59	NS	4.11	0.01	4.44	0.01	5.19	< 0.01
A* B	6	2.61	0.02	1.97	NS	1.35	NS	0.88	NS	1.84	NS	1.31	NS	1.68	NS
	df	S		Fe		Cu		Mn		Zn		B			
		F	Sig.	F	Sig.	F	Sig.	F	Sig.	F	Sig.	F	Sig.		
Land use (A)	3	4.13	< 0.01	1.9	NS	4.07	< 0.01	3.11	0.04	3.98	0.01	0.32	NS		
Topography (B)	2	3.72	0.03	3.88	0.02	10.25	< 0.01	6.19	< 0.01	1.73	NS	23.35	< 0.01		
A* B	6	3.41	< 0.01	1.72	NS	1.07	NS	1.65	NS	0.97	NS	0.46	NS		

NS indicates not significance at p < 0.05.

**Table 5:** Mean surface soil properties (0–20 cm) for land use types (park, n =87; institution, n =131; residential, n =92; roadside, n =95).

Soil properties	Land use				ANOVA *p*
	Park	Institution	Residential	Roadside	
pH	7.70±0.1b	7.81±0.1b	8.15±0.1a	7.93±0.1ab	0.012
Organic matter, g kg^-1^	0.65±0.03	0.64±0.03	0.67±0.06	0.69±0.04	NS ^a^
N, mg kg^-1^	23.57±3.62	25.73±3.14	29.15±6.87	33.13±4.42	NS
P, mg kg^-1^	43.01±4.58a	33.52±8.35ab	27.17±3.82b	35.33±5.37ab	0.013
K, mg kg^-1^	117±6.85	112±5.82	117±12.69	131±8.13	NS
Ca, mg kg^-1^	2407±80b	2394±68b	2634±148ab	2663±96a	0.028
Mg, mg kg^-1^	255±14	234±11	240±26	224±16	NS
S, mg kg^-1^	30.87±2.53b	25.03±2.10b	26.04±4.77b	37.23±3.02a	0.001
Fe, mg kg^-1^	35.11±2.26	32.73±1.86	28.36±4.13	35.12±2.66	NS
Cu, mg kg^-1^	4.64±0.63ab	3.56±0.57b	4.82±1.27ab	6.21±0.73a	0.003
Mn, mg kg^-1^	27.04±1.85a	23.93±1.55ab	20.11±3.46b	27.08±2.13ab	0.035
Zn, mg kg^-1^	5.54±0.66b	6.21±0.55ab	6.47±1.12ab	7.91±0.73a	0.010
B, mg kg^-1^	0.70±0.07	0.73±0.06	0.68±0.12	0.69±0.86	NS

The results are presented as mean ± SD. In a row, values with the same letters are not significantly different at 5% level based on Duncan’s multiple range tests. ^a^ NS, not significant at the α = 0.05 probability level.

Different topographies also exhibited a statistically significant effect for 8 of the 11 chemical variables (p < 0.05, Table 4). Most of the concentrations of macro and micronutrients in plain cities were always higher than those in mountainous cities (Table 6), showing a clear negative correlation with elevation. For N, Ca, Mg, S, Cu, and Mn, differences were nearly 1.5 times as high for the plain cities as for the mountainous cities. Interactions between land use and topography were not significant except for pH and available S concentration (Table 4).

**Table 6:** Mean surface soil properties (0–20 cm) for topography types (hill, n =126; mountain, n =120; plain, n =159).

Soil properties	Physiographic			ANOVA *p*
	Hill	Mountain	Plain	
pH	7.87±0.1ab	7.95±0.01a	7.72±0.01b	0.001
Organic matter, g kg^-1^	0.74±0.03a	0.52±0.04c	0.66±0.03b	0.010
N, mg kg^-1^	25.42±3.51b	21.65±4.82b	34.84±3.16a	0.001
P, mg kg^-1^	37.76±4.32	35.05±5.93	29.17±3.82	NS ^a^
K, mg kg^-1^	121.31±6.65a	98.74±9.02b	128.39±5.98a	0.014
Ca, mg kg^-1^	2327±78b	2160±106b	2839±69a	0.011
Mg, mg kg^-1^	218±14b	178±19c	295±12a	0.005
S, mg kg^-1^	25.06±2.53b	26.93±3.49b	35.44±2.25a	0.027
Fe, mg kg^-1^	28.46±2.11b	31.45±2.92 b	39.74±1.91a	0.019
Cu, mg kg^-1^	5.52±0.61a	2.89±0.84b	4.70±0.55a	<0.001
Mn, mg kg^-1^	18.03±1.80b	12.31±2.48c	40.11±1.68a	0.003
Zn, mg kg^-1^	6.00±0.59	6.82±0.80	6.65±0.52	NS
B, mg kg^-1^	0.95±0.08a	0.69±0.08b	0.48±0.05c	<0.001

The results are presented as mean ± SD. In a row, values with the same letters are not significantly different at 5% level based on Duncan’s multiple range tests. ^a^ NS, not significant at the α = 0.05 probability level.

## Discussion

Urban soils are often subjected to compacting, crushing, sealing, dust, sewage, or alkalizing products; therefore, soil physical biological properties will be affected [18,19]. In this study, urban soil pH, with a mean of 7.88 (Table 3), has clearly deviated from the range found in local plain, hill, or mountain soils (5.0–6.8) [20]. A similar result was observed by Jim [11] in Hong Kong, where only 8% of samples fell below neutrality, and the remainders were alkaline. Chon et al. [21] also found that pH was much higher in roadside soils of Seoul; however, without a noticeable seasonal variation. High pH values are mainly a result of alkalizing products such as calcium carbonate or calcium-magnesium carbonate in gravel, flagstones, cement, concrete, mortar, and atmospheric particulate deposition [4,10,22]. Messenger [23], Short et al. [24], and Pouyat et al. [8] also found that urban soils with construction wastes were alkaline. These alkaline substances become an enduring chemical legacy within the soil profile [11,25]. Leaching, especially by rain or sewage water, can lead to the movement of CaCO_3_ contained in these materials to adjacent intact soils [23]. Elevated soil pH can influence the form and availability of nutrients and can result in plant nutrition imbalances and deficiencies and chlorosis symptoms [4]. Although elevated pH may increase vigor for some alkaline-favoring plant species, it is detrimental to many predominantly humid-subtropical plant species that are adapted to acidic soil.

Soil organic matter is an important part of soil that maintains and protects soil nutrients from environmental loss [22]. In this study, most SOM concentrations were ≤10 g kg^-1^. This is significantly lower than other systems in this region, such as 14.5 g kg^-1^ in agro-ecosystems [26], and 29.8 g kg^-1^ in forest ecosystems [27]. Land preparation generally removes surface plants and topsoil and results in further reductions of SOM [4,22]. Besides these reasons, lower SOM concentration may be related to urban greenland management [3]. In an effort to increase the aesthetic value of cities, workers repeatedly remove fallen leaves, grass clippings and other organic residues, which further reduce organic matter input to soil. City management sectors should improve the management of soil improvement, transportation, and stacking in urban construction to ensure the sustainable use of greenland and soil resources. The stalks and dry leaves, grass clippings, and other organic wastes in cities can be used as a good organic fertilizer when returned to soil [28,29].

The changes of the urban soil environment also affect the effectiveness of soil nutrients, in particular, available N and P. In this study, the available N concentration of 98% samples was below the critical level (50 mg kg^-1^) [7], suggesting that most urban soils in Hubei do not have sufficient N to support garden vegetation growth. Similar results were observed by Baxter et al. [30] in New York City, Jim [11] in Hong Kong, and Zhang et al. [10] in Hefei, which suggests that N deficiency in urban soil is likely to be possibly a common phenomenon. Baxter et al. [30] found that lower soil N concentration may be regulated by the low rates of N mineralization and nitrification in urban soil. Mineralization of N from organic matter in urban soil could be suppressed by the input of poor quality litter or increased amounts of organic matter transferred to recalcitrant pools, or both [30]. White and McDonnell [12] found trampling and high levels of heavy metals in urban areas also indirectly reduced the numbers and diversity of soil microbes and invertebrates, likely leading to low rates of N mineralization and nitrification. Like available soil N, the available soil P was low (mean of 28.3 mg kg^-1^) when compared with the common range (i.e. 30–60 mg kg^-1^) of non-urban soils. Lower available P in urban soil is likely a result of the reduced organic inputs [11,30]. The abundance of carbonates in this region [20] may bind soil P and further limit its availability. The deficiency of both N and P could be rectified by suitable soil improvement measures able to positively affect soil organic matter concentration.

Generally, available K concentration decreases from the north to the south in China primarily due to the higher rainfall and consequent leaching of K in southern China [14]. In the land south of the Yangtze River, farmers need to add K fertilizer for high yields [31]. However, the concentration of available K (125 mg kg^-1^) in urban soil of this region was found to be moderate, slightly higher than the rural average of 79.3 mg kg^-1^ [14]. Similarly, an increase of available K in urban soils has been reported by Hamberg et al. [32]. It is speculated that these results may be related to the high Ca concentration in urban soil, since divalent Ca has higher surface charge densities, and cation exchange interaction, than monovalent K [33]. Consequently, fixed or absorbed K ions from soil colloids could be exchanged with abundant soil Ca, resulting in the higher available soil K.

Compared with macronutrients, the available Ca concentrations of 99% of samples in this study were beyond the recommended range for Ca (Table 3) as were other cities, such as Baltimore (1620 mg kg^-1^) [8] and O’ahu (1280 mg kg^-1^) [34]. Carbonate rock in Hubei is abundant, which has released large amounts of Ca into the soil by weathering [35]. Also, Ca is an important component of atmospheric particulates in urban areas and comes from construction sites where lime, mainly composed of CaO or Ca(OH)_2_, is used [36]. There is much evidence that higher soil Ca concentrations are related to urban construction and road building [36]. Zhang and Iwasaka [36] found that soil Ca concentrations increased along the urban/rural gradient and with increased road density. Suitable increases of Ca in urban soil can be favorable to the cation exchange complex, especially in acid soil, and could remove aluminum toxicity, manganese toxicity, or salt damage [37]. However, excessive Ca would reduce the availability of other nutrients such as B, P, Mo, Fe, and Zn [38,39].

Urban soil had sufficient or excessive micronutrient levels (Mn, Fe, Cu, and Zn), to support plant growth. However, 60% of samples have inadequate soil B supply. The other 40% of samples with high available B may be due to the higher total soil B concentration [40,41]. In addition to the parent material, soil pH was another important factor influencing available soil B concentration [40]. Generally, in peracidic (pH < 3) and peralkaline (pH > 10) soil, available B concentration is low [40]. The reason is that water-soluble B is easily formed and lost in acid soil and that strong absorption to B exists in alkaline soil especially rich in calcium carbonate. However, under subalkaline (pH 7–9) condition, soil available B concentration increases as pH increases [41]. This study verified the above view that, in subalkaline soils, available B concentrations in mountainous and hilly cities with high pH was higher than that in plain cities with low pH.

### Effect of Urban Land Use

Different environments and degrees of human disturbance in different land use areas induced great changes in some soil physical and chemical properties [22,42]. Pouyat et al. [8] reported that K, P, pH, and bulk density in urban forest (undisturbed and unmanaged) were significantly lower than in institutional and residential areas (disturbed and managed), suggesting that human disturbance and management increased soil nutrients (particularly P and K from fertilizers). In this study, soil pH and available P concentration significantly differed between land use types, with the highest values for pH and lowest concentrations for available P in the residential types and the lowest values for pH and highest concentrations for available P in the park sites. As the initial disturbances of land development often incorporate many alkaline substances such as liming and cement and elevated soil pH can influence the form and availability of nutrients, the difference between land use types may be due to the extent of surface site disturbance. Park soils are generally minimally disturbed and have no fertilizer management while road, institution, and residential soils are impacted by human trampling, mechanical compression, incorporation of human sewage, and building materials. Urban soil nutrients were also likely to be influenced by dust precipitation, because dust could provide a rich source of Ca, Mg, and S [43] and their concentrations are significantly different in each landscape, with the road site having the highest concentration of dust [21,37]. In this study, the roadside site had significantly higher concentrations of available soil Ca and S than the institutional and park sites, but available Mg was not significantly different (p > 0.05) among the four land use types. Most soils in this region are derived from dolomite, which is rich in Mg. In contrast, dust contains small amounts of Mg, and hence likely did not lead to the differences among land use types. In this study, available Cu, Mn, and Zn significantly differed among the four land use types, with the road sites having the highest of concentrations of Cu, Mn, and Zn. It is speculated that the higher soil temperature at the road sites resulted in higher soil mineralization and higher nutrient availability [44]. Oke [45] reported that the introduction of asphalt of urban soil surfaces could increase the absorption and storage of solar energy that later is released as sensible heat, which increases adjacent soil temperatures by as much as 3–5°C. An increase in the content of available nutrients such as Cu, Mn, and Zn of road sites caused by high temperature was greatly beyond the maximum threshold values of these elements. This may explain why plants along the roads in some areas are more prone to leaf yellowing phenomenon or fails of plant establishment than in other land use sites. So, it should be choose some plants that are not sensitive to Cu, Mn, and Zn at the road sides.

### Effect of Topography

Large differences in urban soil nutrients existed among cities on the three topographies (p < 0.01; Table 6). The concentrations of N, K, Ca, Mg, S, Fe, Cu, and Mn in plain cities were higher than those in mountainous cities, showing a negative correlation with city elevation. Pouyat et al. [8] found soil chemical and physical properties differed between geological provinces; however, lower values corresponded to the Atlantic Coastal Plain and higher values to the Piedmont Plateau. These differences are possibly caused by differences in the elemental composition of parent material between cities with different elevations. In this study, urban soils were yellow-brown, mainly derived from water-transported alluvium or formed by in-situ weathering of rocks (carbonate and sandshale), and easily suffered nutrient leaching during the rainy season. Soil in higher terrain was more vulnerable to leaching of available soil nutrients than in relative lower terrain because of the loss of soil organic matter [14,46].

Differences in temperature along the topographies may be the second reason for differences in available nutrients between cities of different elevations. Temperature in most plain cities was higher than in mountainous cities [47]. Curtin et al. [44] found higher soil temperatures to significantly increase soil C and N mineralization and solubility of available soil nutrients. Li et al. [48] also reported the concentrations of available soil N and P increased by ~50% and 10%, respectively when the temperature increased from 12 to 18°C. Higher temperatures in plain cities likely increased soil nutrient mineralization and its availability.

Dust is likely to be another reason for the difference in soil nutrients as it is a major source of nutrient input [21]. Based on a dust study in Hubei, mountainous urban environments usually had lower concentrations and depositional fluxes of atmospheric dust than plain urban environments [49]. Urbanization is often more rapid in plain areas because road alignment is relatively easy and construction costs are also lower. Increased air pollution (providing soil input of N, Ca, Mg, and S) in plain areas likely further increased soil nutrients compared with those in mountainous areas.

## Conclusion

In general, urban soils in Hubei Province had a higher pH than natural soils, were deficient in organic matter, and low in available N, P, and B concentrations. Concentrations of Ca, Mg, and S, however, were sufficient in many areas, particularly along roadside and residential land use types. This study indicates that these nutrient properties are highly variable across urban land uses and topographies, suggesting that urban soils are diverse, complex, and affected by numerous management practices, disturbances, and environmental factors. Future research should examine urban soil properties in various spatial and temporal scales. This would provide more specific information about the spatial and temporal variability in urban soils, to develop appropriate techniques and strategies for future urban design and planning.
